# Degradation of Organic Matter in Sauce-Flavored Liquor Wastewater by Catalytic Oxidation Performance of Mn_2_Cu_2_O_x_/Al_2_O_3_ Catalysts in Treatment and Mechanism Research

**DOI:** 10.3390/molecules30061242

**Published:** 2025-03-10

**Authors:** Benfu Luo, Jie Yu, Weiwei Huang, Xuanyu Zhou, Jinyin Li, Yuhang Liu, Xi Yang, Xiang Zhou, Haiyan Ning, Yujing Yan, Haixing He

**Affiliations:** 1 School of Architecture and Civil Engineering, Xihua University, Chengdu 610039, China; y540751@163.com (J.Y.); sshuang0815@163.com (X.Z.); 117671290047@163.com (Y.L.); petrel@mail.xhu.edu.cn (H.N.); yooho666@outlook.com (Y.Y.); hhxznb@163.com (H.H.); 2 China Municipal Engineering Zhongnan Design and Research Institute Co., Ltd., Wuhan 430010, China; hww1010@foxmail.com (W.H.); ljy_1102@163.com (J.L.); 3 Chengdu University Library, Chengdu University, Chengdu 610106, China; xiyang151503@163.com; 4 Suyi Design Group Co., Ltd., Nanjing 210012, China; xuzhu719@126.com

**Keywords:** Mn_2_Cu_2_O_x_/A_l2_O_3_ catalyst, sauce-flavored liquor wastewater, catalytic oxidation, mechanism study

## Abstract

With the rapid growth of the sauce-flavored liquor industry, the treatment of wastewater has become an increasingly critical challenge. This study seeks to assess the catalytic oxidation efficacy of Mn_2_Cu_2_O_x_/Al_2_O_3_ catalysts in the degradation of organic pollutants present in sauce-flavored liquor wastewater, while also elucidating the mechanisms underpinning their performance. Mn_2_Cu_2_O_x_/Al_2_O_3_ catalysts were synthesized, and their physicochemical properties were thoroughly characterized using advanced techniques such as Brunauer–Emmett–Teller (BET) analysis, N_2_ sorption isotherm analysis, scanning electron microscopy (SEM), and X-ray photoelectron spectroscopy (XPS). Moreover, the key active species involved in the catalytic oxidation process, including hydroxyl radicals (•OH) and superoxide anion radicals (•O_2_^−^), were identified through hydroxyl radical quenching experiments employing tertiary butyl alcohol (TBA). The contribution of these free radicals to enhancing the ozone catalytic oxidation performance was also systematically evaluated. Based on both experimental data and theoretical analyses, the Mn_2_Cu_2_O_x_/Al_2_O_3_ catalysts demonstrate remarkable catalytic activity and stability, significantly reducing chemical oxygen demand (COD) levels in wastewater. Furthermore, the catalysts are capable of activating oxygen molecules (O_2_) during the reaction, producing reactive oxygen species, such as •O_2_^−^ and •OH, which are potent oxidizing agents that effectively decompose organic pollutants in wastewater. The proposed catalysts represent a highly promising solution for the treatment of sauce-flavored liquor wastewater and lays a solid foundation for its future industrial application.

## 1. Introduction

In recent years, the sauce-flavored liquor industry has entered a phase of rapid growth. However, managing the wastewater generated during sauce-flavored liquor brewing has emerged as a significant environmental challenge. This wastewater is characterized by its high concentration of organic matter, exceptionally elevated COD, substantial production volume, and complex composition. As a result, it is classified as a highly concentrated organic wastewater that is challenging to degrade [1,2].

Sauce-flavored liquor industrial wastewater undergoes a combination of advanced treatment processes, including front-end anaerobic, middle-stage biochemical, and back-end coagulation, oxidation, and even membrane filtration. The COD retains a high concentration of pollutants. Consequently, relying solely on conventional “anaerobic + aerobic” processes is insufficient in effectively removing these hard-to-degrade substances. Therefore, employing advanced treatment technologies is essential to enhance the treatment efficiency of the biochemical effluent. Currently, the primary methods for reducing the COD in sauce-flavored liquor wastewater include activated carbon adsorption, coagulation–precipitation, and Fenton oxidation [3]. However, both activated carbon adsorption and coagulation–precipitation fail to completely degrade refractory organics and result in secondary pollution due to sludge generation [4]. Although Fenton oxidation effectively treats such wastewater, it requires a significant amount of chemicals, making the process expensive. Ozone oxidation demonstrates high reaction efficiency for removing pollutants in wastewater and does not produce secondary pollution [5]. However, studies and practical applications [6] have revealed that ozone oxidation alone is insufficient to meet the discharge standards for sauce-flavored liquor wastewater due to its selectivity, low utilization efficiency, inadequate oxidative capacity, and insufficient ozone content. To address these limitations, catalytic ozone oxidation has emerged as a promising approach for degrading organic matter in sauce-flavored liquor wastewater. Research indicates that while various commercially available catalysts have been tested for reducing the COD in this type of wastewater, they often exhibit significant drawbacks, including high costs, restrictions due to intellectual property rights, and high metal element loading. Therefore, it remains essential to develop ozone-catalyzed oxidation methods tailored to the complex and dispersed nature of sauce-flavored liquor wastewater; this includes optimizing catalyst preparation and activation, determining the optimal ozone-to-catalyst ratio, evaluating catalytic performance, and investigating the underlying reaction mechanisms.

Studies have demonstrated that the catalytic oxidation performance of homemade Mn-Cu/Al catalysts, particularly those with a ratio of wt%2: wt%2 in the Mn-Cu/Al class, represents a highly effective approach for degrading organic matter in soy sauce wastewater. This method balances catalytic efficiency, cost effectiveness, and catalyst stability. Multiple studies have verified the efficacy of Mn-Cu/Al catalysts in catalytic oxidation processes. Ye Jiang et al. [7] employed a homemade Mn_5Cu_1 catalyst to catalytically oxidize toluene, leveraging the synergistic interactions between Mn and Cu. The results highlighted its excellent catalytic oxidation performance. Similarly, Mengxue Yin et al. [8] prepared Zn-Cu/Al catalysts using the impregnation method and evaluated their desulfurization performance at low temperatures. Their findings revealed that Zn_3Cu_3 catalysts achieved the highest penetration capacity of 353.91 mg/g at 50 °C and 50% relative humidity, demonstrating remarkable desulfurization efficiency. Furthermore, Zhixing Li et al. [9] synthesized Mn-Fe/ZSM-5 catalysts via the impregnation method to catalyze ozonation and degrade nitrobenzene (NB) in wastewater. Under optimal experimental conditions, the removal efficiencies of NB and total organic carbon (TOC) reached 99.99% and 72%, respectively, within 40 min.

In this study, γ-alumina was selected as the catalyst carrier due to its stable structural properties, porous nature, high specific surface area, and excellent adsorption capabilities. Mn and Cu, with a ratio of wt%2: wt%2, were loaded onto the γ-alumina carrier as active components using the impregnation method. Actual sauce-flavored liquor wastewater was used as the test water sample, and the developed catalyst was incorporated into the ozone reaction column, where ozone was introduced to evaluate its effectiveness in the advanced treatment of soy sauce liquor wastewater. This study aimed to assess the characteristics and performance of the catalyst, determine the optimal reaction conditions and kinetic equations, and investigate the reaction mechanism. Furthermore, the potential of heterogeneous catalytic ozonation technology for practical applications was explored, highlighting its viability for real-world implementation in wastewater treatment.

## 2. Results and Discussion

### 2.1. Catalyst Characterization

BET surface area analysis, SEM electron microscopy imaging, and XPS inspections were conducted on both γ-Al_2_O_3_ and Mn_2_Cu_2_O_x_/Al_2_O_3_ catalysts. The alterations in specific surface area, pore volume, and pore size of the γ-Al_2_O_3_ support following high-temperature calcination were assessed.

**Figure 1:**
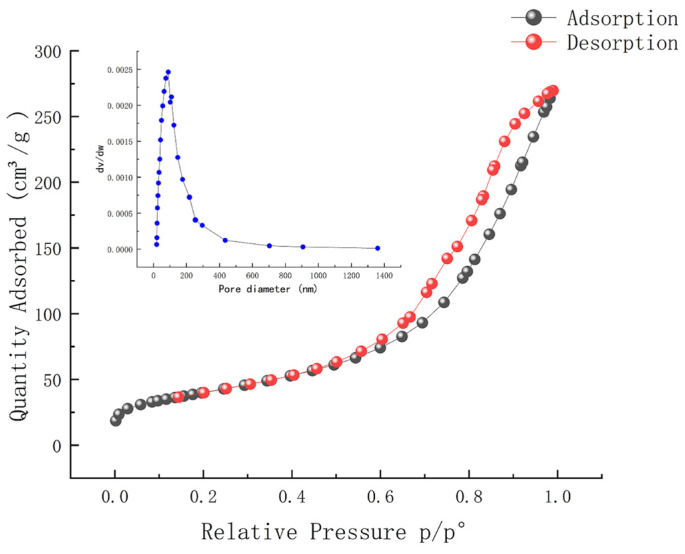
N_2_ adsorption–desorption isotherms and pore size distribution results.

The BET results in Table 1 indicate that the crystal structure of γ-Al₂O₃ undergoes changes after calcination at a high temperature of 600 °C. During this process, the small pores in the material merge, or some of the pore structures are rearranged, and certain impurities, such as adsorbed water and organic compounds, are removed, leading to an increase in the specific surface area. After the loading of active substances onto γ-Al_2_O_3_, the incorporation of metals such as Mn, Cu, and Ce may result in catalyst pore closure or a more compact arrangement. These metals may be deposited onto the surface of γ-Al_2_O_3_, forming metal particles or interacting with the alumina, thereby altering the pore structure and causing a reduction in the specific surface area after loading. The structural analysis of the catalysts after different treatments, as well as the results of N_2_ adsorption-desorption isotherms and pore size distributions, are shown in Table 1 and Figure 1.

**Figure 2:**
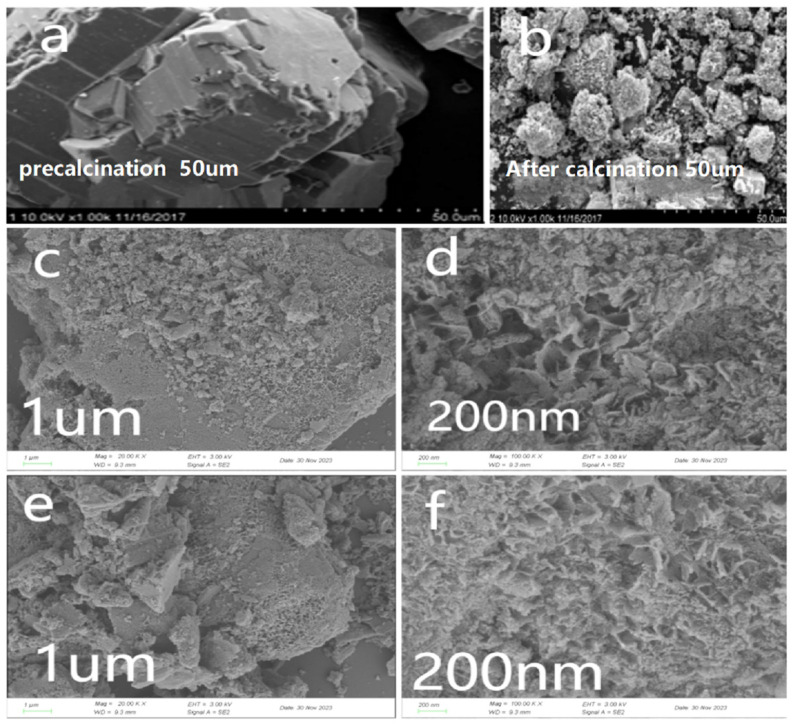
SEM image of homemade γ-Al_2_O_3_ catalysts: (**a**,**b**) γ-Al_2_O_3_ (before calcination, after calcination); (**c**,**d**) Mn-Cu/Al (wt%2: wt%2); (**e**,**f**) Mn-Cu-Ce/Al (wt%2: wt%2: wt%2).

The SEM image analysis in Figure 2 presented in the figure reveals that γ-Al_2_O_3_ exhibits irregularly sized particles with rough surfaces after calcination. The voids between these particles provide ample space for the subsequent loading of active components. Following the loading process, γ-Al_2_O_3_ shows a significant reduction in surface depressions and pores, suggesting a more uniform distribution of the active ingredients on the carrier surface.

**Figure 3:**
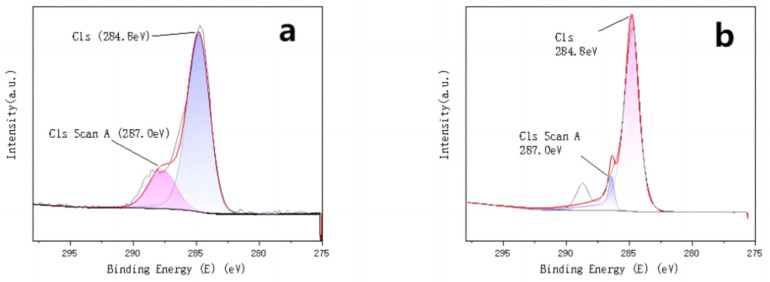
γ-Al_2_O_3_ (**a**) and Mn Cu/γ- peak fitting diagram of C element in Al_2_O_3_ (**b**).

**Figure 4:**
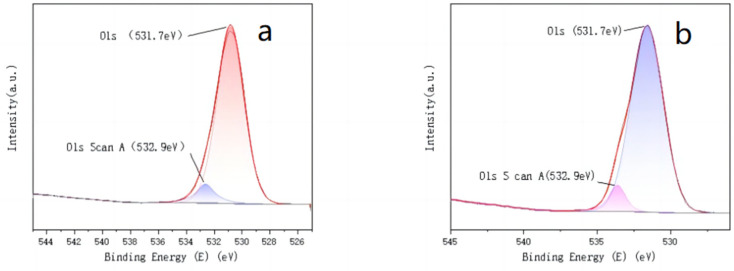
γ-Al_2_O_3_ (**a**) and Mn Cu/γ- peak fitting diagram of O element in Al_2_O_3_ (**b**).

**Figure 5:**
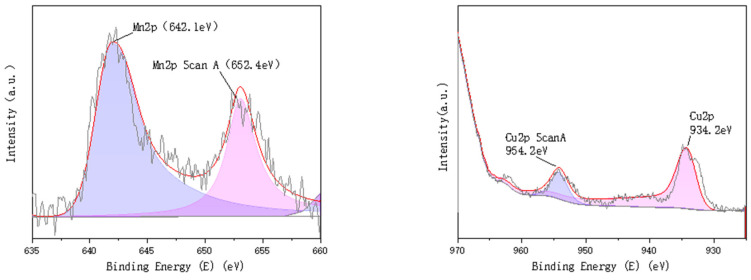
Mn Cu/γ- peak fitting diagram of Mn and Cu element in Al_2_O_3_.

To compare the chemical valence and compositional changes of the two catalysts before and after the loading of active components, XPS characterization was performed on both catalysts. The characterisation results are shown in Figure 3, Figure 4 and Figure 5.

The analysis of the fitted peaks for the C and O elements, before and after loading the Mn-Cu/Al catalysts, reveals minimal changes in the C element. Both spectra are primarily characterized by the C-C bond at 284.8 eV, with a minor presence of C-O functional groups at 287.0 eV [10]. Similarly, there is little variation in the O element before and after loading, with two identical fitted peaks corresponding to O = C/O = S (531.7 eV) and O-C/O-S (532.9 eV) in both the C and D panels [11,12]. The Mn element exists in two valence states, Mn^2+^ and Mn^3+^, after loading onto the γ-Al_2_O_3_ carrier [13]. The two corresponding fitted peaks are located at 642.1 eV and 652.4 eV, respectively. Both Mn (III) and Mn (II) exhibit strong catalytic activity for the non-homogeneous ozone-catalyzed oxidative degradation of organic compounds [14,15]. The Cu element in the homemade Mn-Cu/γ-Al_2_O_3_ catalysts primarily exists in the Cu^2+^ and Cu^+^ valence states [16,17].

### 2.2. Kinetic Analysis of Catalytic Oxidation Reactions

**Figure 6:**
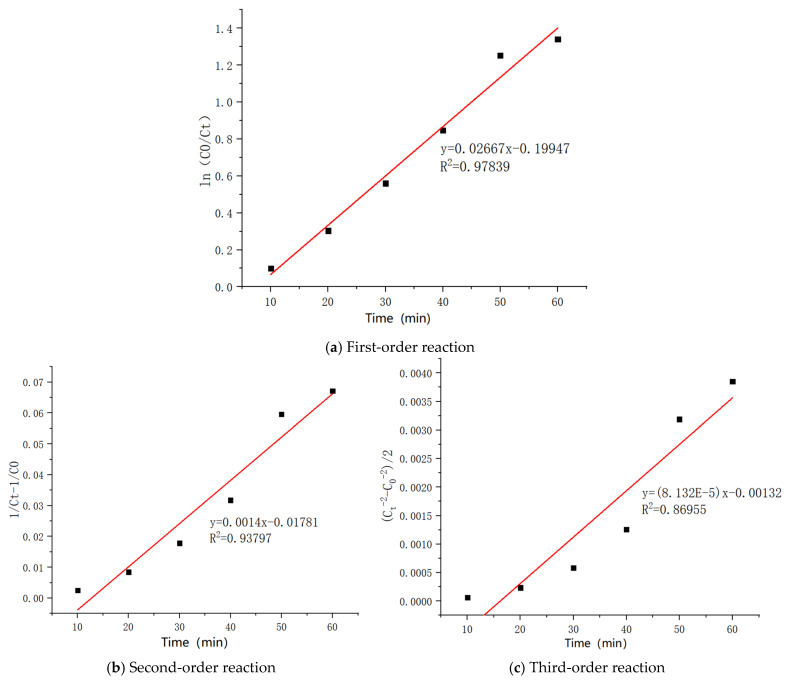
Linear regression results of the order of heterogeneous ozone catalytic oxidation reaction.

The experimental results for the homemade non-homogeneous ozone catalyst under optimal conditions were analyzed by fitting first-order, second-order, and third-order reaction kinetics, as shown in Figure 6.

To evaluate the appropriateness of various kinetic models for the experimental data and determine the most suitable model for characterizing the reaction process, an error analysis was conducted to assess the kinetic behavior of the first-order, second-order, and third-order reactions of the heterogeneous-phase ozone catalysts under optimal conditions.

Based on the data presented in Table 2, the standard deviation was computed to be 0.01498, which is relatively small, suggesting that the experimental data are reproducible and the fitting results are reliable.

From the linear regression plot of the reaction order, it is evident that the highest correlation coefficient (R^2^) corresponds to the first-order reaction, with an R^2^ value of 0.97839. This indicates the best fit between non-homogeneous ozone and first-order reaction kinetics. Therefore, the homemade Mn-Cu/Al catalyst (wt%2: wt%2) is most suitable for the first-order reaction kinetics model when applied in the non-homogeneous ozone degradation of COD in sauce-flavored liquor wastewater. The equation represents the concentration of the reactants as a function of time, where C_0_ is the initial concentration, Ct is the concentration at time t, and 0.02667 is the reaction rate constant, k, which denotes the rate of the reaction.

(1) $$ln\frac{C_{0}}{C_{t}}=0.02667t-0.19947$$

### 2.3. Reaction Mechanism Study

The catalytic oxidative degradation of organic matter in wastewater involves the oxidative removal of organic contaminants through the enhanced decomposition of ozone, resulting in the generation of additional hydroxyl radicals (•OH) in the presence of a catalyst.

#### 2.3.1. GC-MS Water Sample Detection

The water samples, both before and after treatment, were analyzed using gas chromatography–mass spectrometry (GC-MS) for comparative analysis. The GC-MS results in Table 3 and Table 4 indicate that the non-homogeneous ozone catalytic oxidation process effectively oxidizes organic compounds such as sugars, water-soluble macromolecules, and aromatics. However, the treatment is less effective on aliphatic compounds and saturated fatty carbonyl compounds. Notably, certain aliphatic compounds were still present in the water samples following treatment.

(2) $$CODconversion\;factor=\frac{(carbon\;atom\;number\times 2+hydrogen\;atom\;number\times 0.5-atomic\;number\;of\;oxygen)\times 16}{molecular\;mass}$$

#### 2.3.2. •OH Quenching Experiments

**Figure 7:**
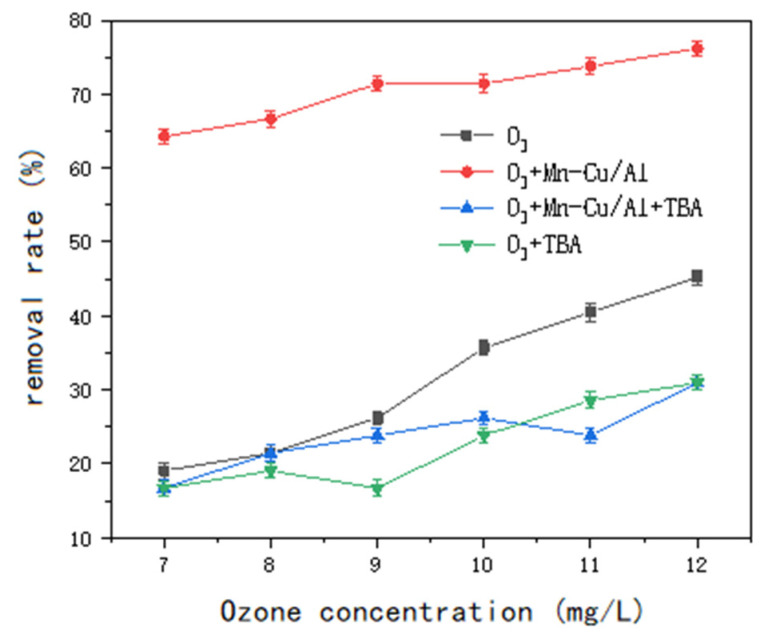
Research on the COD degradation rate of TBA.

•OH is considered the primary reactive oxygen species (ROS) in ozone oxidation [18]. When tert-butanol is introduced into the system, it acts as a highly efficient quencher of hydroxyl radicals. As a result, the hydroxyl radicals rapidly react with and are consumed by the tert-butanol, effectively terminating the chain reaction initiated by radicals during ozone oxidation. Thus, the presence of hydroxyl radicals in the reaction can be inferred based on the addition of tert-butanol. In this experiment, the water sample had a COD of 42 mg/L, and the tert-butanol dosage was 20 mmol/L. The impact of free radicals on the ozone catalytic oxidation performance was assessed by comparing the COD removal rate across different groups. The results are presented in Figure 7.

From the comparison of the data in the figure, it is evident that the addition of TBA did not significantly affect the COD removal rate in ozone-only oxidation when the ozone concentration was below 10 mg/L. In this case, ozone alone exhibited poor effectiveness in COD degradation. However, in the non-homogeneous system of O₃ + Mn-Cu/Al, the removal rate decreased markedly upon the addition of the quenching agent TBA. This suggests that a large number of hydroxyl radicals were generated during the reaction in the presence of O₃ + Mn-Cu/Al, and the oxidation of organic matter was primarily driven by hydroxyl radicals. Therefore, in the non-homogeneous ozonation process for COD degradation in sauce-flavored liquor wastewater, the indirect oxidation pathway via hydroxyl radicals (•OH) plays a dominant role throughout the reaction.

#### 2.3.3. Analysis of Reaction Mechanisms

The catalytic oxidation mechanism is generally understood as the chemical adsorption of ozone on the catalyst surface, leading to the formation of reactive species that subsequently degrade organic matter [19].

**Figure 8:**
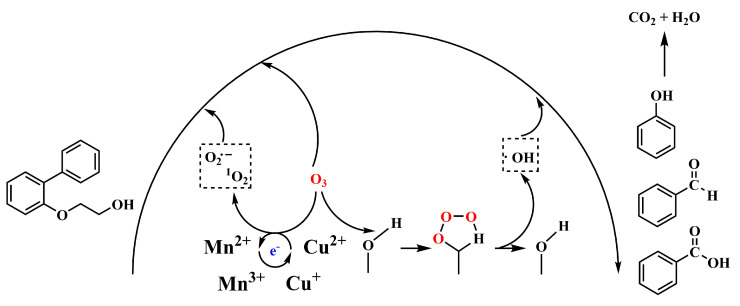
Mechanism diagram of Mn Cu/Al-catalyzed ozone degradation of organic compounds.

As illustrated in Figure 8, the degradation of 2-([1,1′-biphenyl]-2-yloxy)ethanol (C_14_H_14_O_2_) proceeds with the formation of several intermediate compounds, such as phenol, benzaldehyde, and benzoic acid, culminating in the production of CO_2_ and H_2_O under oxidative conditions facilitated by •OH.

The catalyst also facilitates the activation of oxygen molecules (O_2_) during the reaction process, leading to the generation of reactive oxygen species such as the superoxide anion (O_2_^−^) and the hydroxyl radical (•OH). The presence of these ROS plays a crucial role in the decomposition of ozone on the catalyst surface [20]. The O_3_ molecules then diffuse from the liquid phase to the catalyst surface, where they form a five-membered ring structure with the hydroxyl groups on the catalyst surface, as depicted in Figure 8. The O_3_ molecules subsequently undergo bond relaxation through interactions with the surface hydroxyl groups, which facilitates electron transfer and the formation of an active species hydroperoxyl radical (HO_2_^−^) on the catalyst surface, accompanied by the release of oxygen. The generated surface (HO_2_^−^) then continues to react with O_3_ molecules, generating ozonide (O_3_^−^) or a hydroxyl radical anion (HO_3_^−^) on the catalyst surface. Subsequently, hydroxyl radicals (•OH) are produced in the system via two pathways: either through the self-decomposition of the HO_3_^−^ to release O_2_ or through mutual reactions between O_3_^−^ and HO_3_^−^. Ultimately, the hydroxyl radical (•OH) dominates the oxidation of organic compounds, leading to their complete mineralization to CO_2_ and H_2_O. At the end of the reaction, the catalyst’s active site adsorbs newly activated water molecules, forming surface hydroxyls, thereby initiating the next catalytic cycle.

Manganese and copper in Mn-Cu/Al catalysts exist in various valence states. For instance, manganese is present in two forms, Mn^2+^ and Mn^3+^, and the valence of manganese ions in MnOx on the catalyst surface may shift, facilitating the electron transfer to ozone. The greater the number of oxygen vacancies, the greater the catalyst’s ability to adsorb and decompose ozone [21], thereby explaining the superior catalytic performance of metal oxide-loaded catalysts.

4Mn^n+1^ + 2O_b_^2−^ → 4Mn^n+^ + O_2_ (3)

2O_3_ + 4e^−^ → 2O_b_^2−^ + 2O_2_ (4)

where O_b_^2−^ is lattice oxygen.

Throughout the ozone catalytic oxidation process, the continuous redox cycle between Mn^n+^ and Cu^n+^ results in a constant overflow of electron transfer, which leads to the creation of additional oxygen vacancies on the Mn-Cu/Al catalyst surface, thereby enhancing its catalytic performance.

In Mn_2_Cu_2_O_x_/Al_2_O_3_ catalysts, the oxides of Mn and Cu form a heterogeneous structure in conjunction with Al_2_O_3_ carriers, effectively promoting charge separation and transfer. The presence of a built-in electric field between the Mn and Cu oxides facilitates the rapid separation and migration of photogenerated electrons and holes to the catalyst surface, thereby enhancing the generation of reactive radicals such as hydroxyl and superoxide radicals. The synergistic interaction between Mn and Cu significantly improves the catalyst’s degradation efficiency, as the oxides of Mn and Cu may participate in the degradation reaction through distinct pathways, leading to more efficient degradation. Moreover, this synergistic effect contributes to the catalyst’s stability, preventing deactivation during the reaction.

## 3. Materials and Methods

### 3.1. Oxidation Performance Test of Commercially Available Catalysts

Ten commercially available non-homogeneous ozone catalysts were selected for this study, comprising three aluminum-based catalysts (A, B, and C), two ceramic-based catalysts (D and E), three iron–carbon catalysts (F, G, and H), and two activated carbon catalysts (I and J).

**Figure 9:**
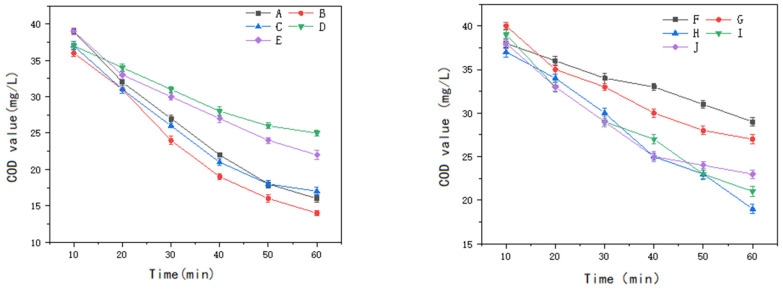
Comparison of catalytic effects of 10 commercially available heterogeneous ozone catalysts.

The experiments were performed using wastewater with a COD of 42 mg/L, an initial pH of 8, an ozone inlet concentration of 10 mg/L, an ozone flow rate of 0.5 L/min, and a catalyst dosage of 500 g per 3 L of water. The catalytic performance of each catalyst in degrading the COD of the sauce-flavored liquor wastewater is illustrated in Figure 9.

### 3.2. Catalyst Preparation

#### 3.2.1. Raw Material Selection

In this study, non-homogeneous ozone catalysts were synthesized via the impregnation method. γ-Alumina was selected as the support due to its stable structural properties, porous nature, high specific surface area, and excellent adsorption capacity [22]. Ultrapure water was employed to pre-treat the support, effectively eliminating impurities and optimizing the pore structure and surface properties, thereby enhancing the impregnation efficiency. The catalyst impregnation precursors, Mn(NO_3_)_3_, FeSO_4_·7H_2_O, Cu(NO_3_)_2_·3H_2_O, and Ce(NO_3_)_3_·6H_2_O, were dissolved to prepare the impregnation solution, allowing the active components to be loaded onto the support surface or into its pores via adsorption.

#### 3.2.2. Preparation Methods

The preparation procedure consisted of four steps, as outlined below:

Step 1: Carrier cleaning and activation. The γ-alumina particles (with a particle size of 2–4 mm) were washed with ultrapure water until all surface dust was completely removed. The material was then subjected to roasting at 600 °C for 4 h and subsequently allowed to cool.

Step 2: Calculation of the water absorption rate. A 100 g sample of the cooled γ-alumina support material was placed in a beaker, and ultrapure water was added until the support was fully immersed. After 12 h, the support was removed and weighed, and the mass after immersion was subtracted from the initial 100 g. The difference represents the water absorption of the support. The calculation is provided in Equation (5) below:

(5) $$W=\frac{m_{after}-m_{before}}{m_{before}}\times 100\%$$

where W is the water absorption, %; m (after) is the mass of the carrier after water absorption, g; and m (before) is the mass of the carrier before water absorption, g.

Step 3: Impregnation of active ingredients. An appropriate volume of ultrapure water was added to a beaker. Once the impregnation solution was prepared, the carrier γ-alumina was introduced and subjected to impregnation for 12 h at room temperature.

Step 4: Drying and calcination. The impregnated carriers were placed in a constant-temperature drying oven, where they were dried at 80 °C for 6 h. Subsequently, the carriers were transferred to a muffle furnace and calcined at 600 °C for 4 h. After calcination, the samples were removed and set aside.

In this experiment, a total of 17 catalysts with varying loading ratios were synthesized according to the aforementioned procedure (refer to Table 5). The most effective metal oxide catalysts and their corresponding loading ratios for non-homogeneous ozone catalytic oxidation were compared and selected.

### 3.3. Oxidation Performance Test of Homemade Catalysts

#### 3.3.1. Effect of Type of Homemade Catalyst on Treatment Effectiveness

Four ozone reaction columns were utilized, each containing 3 L of wastewater and 500 g of catalyst. The second batch of water samples, with a COD value of 42 mg/L, was used as the test water.

**Figure 10:**
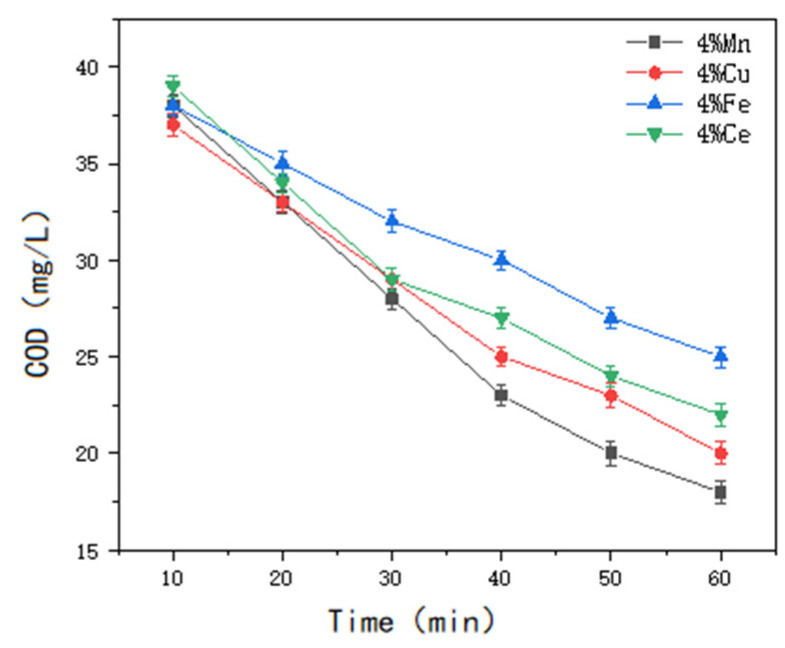
The influence of single metal-supported catalyst types on the heterogeneous ozone treatment efficiency.

**Figure 11:**
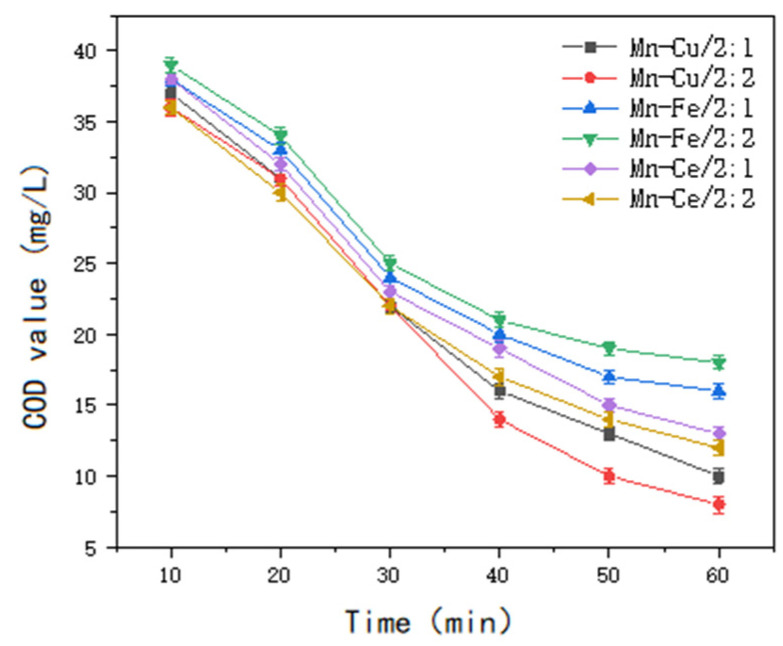
The influence of two types of metal-supported catalysts on the heterogeneous ozone treatment efficiency.

**Figure 12:**
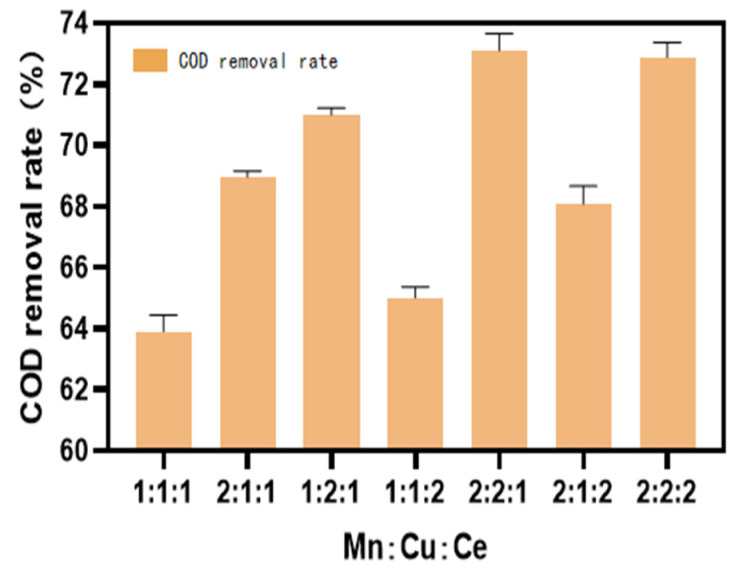
The effect of catalysts prepared with different ratios of Mn, Cu, and Ce on treatment efficiency.

The COD values were measured after a reaction period of 60 min, with additional measurements taken at specified intervals to assess the influence of different non-homogeneous catalyst types on the removal of COD from soy sauce wastewater. The results are presented in Figure 10, Figure 11 and Figure 12.

The results indicated a significant improvement in the COD degradation performance with single-metal-loaded catalysts; However, the treatment effect still did not meet the required standard. When the catalysts were loaded with two metal ions, the Mn-Cu/Al catalysts, with a ratio of wt%2: wt%2, exhibited the best treatment performance. For the Mn-Cu-Ce/Al catalysts, which were prepared by loading three types of metals, the Mn-Cu/Al catalysts with a ratio of wt%2: wt%2 were selected, considering factors such as experimental efficacy, cost effectiveness, catalyst stability, and other variables.

#### 3.3.2. Influence of Catalyst Dosage on Treatment Effect

**Figure 13:**
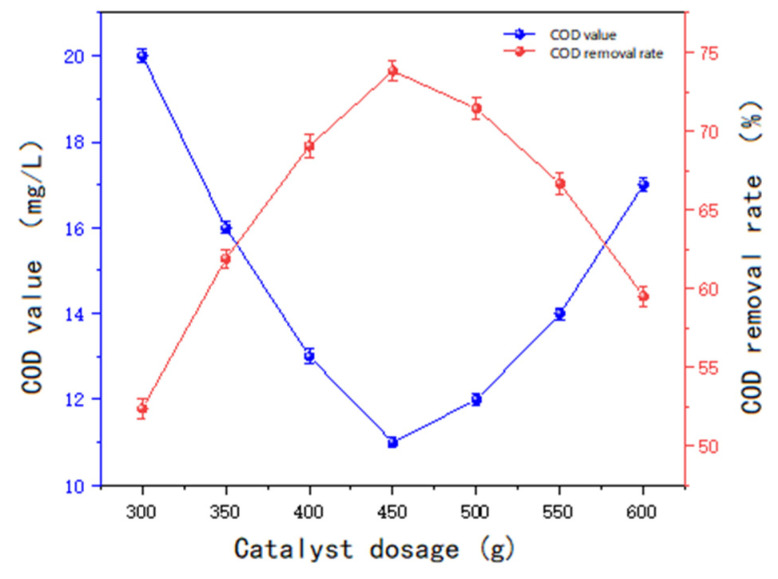
The effect of catalyst dosage on treatment efficiency.

The experimental procedure followed the same steps as outlined previously. The initial parameters were set as follows: pH = 8, ozone inlet concentration = 8 mg/L, and inlet flow rate = 0.5 L/min. The catalyst dosage was varied in the following gradient: 300 g, 350 g, 400 g, 450 g, 500 g, 550 g, and 600 g. After a 60 min reaction period, the COD values of the water samples were determined. The experimental results are presented in Figure 13.

**Figure 14:**
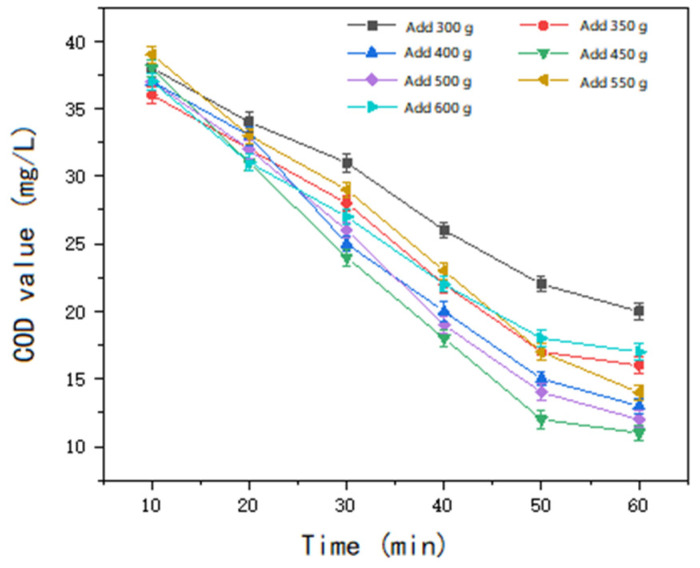
Changes in COD over time under different dosages.

As illustrated in Figure 13 and Figure 14, the treatment efficiency of the non-homogeneous ozone becomes progressively more pronounced with increasing catalyst dosage. The optimal effect is achieved when the catalyst dosage reaches 450 g. Beyond this point, increasing the dosage to 500 g does not yield a significant improvement in treatment efficacy. Further increases in catalyst dosage lead to a noticeable decline in the removal rate. As the catalyst dosage increases, the number of reactive oxygen species (ROS), such as hydroxyl radicals (OH), generated in the reaction system also increases. However, when the catalyst dosage exceeds a certain threshold, the excess radicals may undergo mutual quenching reactions, reducing the effective utilization of these radicals and thereby diminishing the oxidizing capacity for organic matter degradation. Furthermore, excessive catalyst amounts may lead to the accumulation of catalyst particles, which can clog the pore structure of the reactor, ultimately reducing reaction efficiency. Consequently, the experimental results indicate that a catalyst dosage of 450 g for 3 L of water (150 g/L) is optimal for the non-homogeneous ozone degradation of COD in sauce-flavored liquor wastewater.

#### 3.3.3. Catalyst Stability Tests

To evaluate the oxidation stability of the catalysts, the Mn-Cu/Al non-homogeneous catalysts were selected for multiple sequential batch reproducibility tests. Each test utilized 3 L of water, with a single addition of 450 g of catalyst and a reaction time of 60 min.

**Figure 15:**
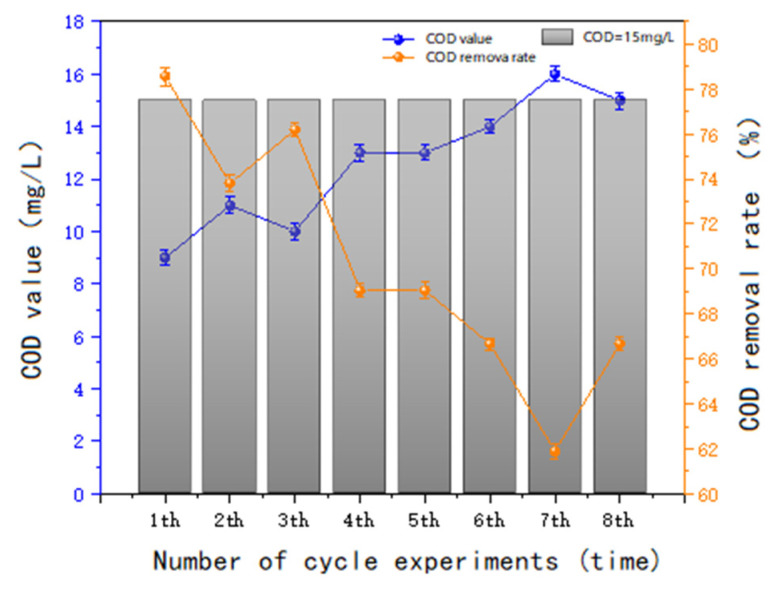
COD removal after cyclic use of Mn Cu/Al catalyst.

From Figure 15, it can be concluded that after sequential batch repetition, the Mn-Cu/Al catalyst demonstrated a gradual decrease in its COD removal efficiency for sauce-flavored liquor wastewater. However, the removal rate remained stable at or above 66.67%. This suggests that the novel, homemade Mn-Cu/Al catalyst exhibits favorable stability and is effective in degrading the COD of sauce-flavored liquor wastewater, highlighting its practical applicability as a catalyst.

## 4. Conclusions

Traditional ozone-only oxidation technology for treating sauce-flavored liquor wastewater, although somewhat effective, has several limitations, including excessive ozone consumption, low ozone utilization efficiency, environmental concerns, and resource wastage. Additionally, commercially available catalysts for degrading COD in sauce-flavored liquor wastewater are less effective, suffering from high costs, intellectual property restrictions, and a large metal loading. This study proposes the use of γ-alumina as a support to prepare Mn_2_Cu_2_O_x_/Al_2_O_3_ catalysts via the impregnation method for the catalytic oxidation of sauce-flavored liquor wastewater. The results demonstrate that the catalyst effectively removes the COD with good stability, primarily relying on hydroxyl radical oxidation, a process involving the generation and activation of active oxide species. The kinetic analysis of the homemade Mn-Cu/Al catalysts for heterogeneous ozone catalytic oxidation reveals a first-order reaction rate. Both Mn and Cu loaded on the support exhibit strong catalytic activity, and their synergistic effect significantly enhances oxidation efficiency. The Mn_2_Cu_2_O_x_/Al_2_O_3_ catalyst presents a promising option for sauce-flavored liquor wastewater treatment, offering valuable insights into catalytic oxidation technology and laying the groundwork for future industrial applications.

## Figures and Tables

**Table 1:** BET specific surface area and pore structure analysis of catalysts.

	Performances	Specific Surface Area (m^2^/g)	Pore Volume (cm^3^/g)	Pore Size (nm)
Catalysts				
γ-Al_2_O_3_ (no treatment)		201.50	0.40	8.30
γ-Al_2_O_3_ (600 °C, 4 h)		236.10	0.68	12.80
Mn-Cu/Al (Mn: Cu = 2:2)		145.03	0.43	11.84
Mn-Cu-Ce/Al (Mn: Cu: Ce = 2:2:2)		140.56	0.42	11.46

**Table 2:** Error analysis.

Response Order	Reaction Rate Constant k	Correlation Coefficient R^2^	Deviation	Deviation Squared
First order	0.02667	0.97839	0.017286	0.0002988
Second order	0.0014	0.93797	−0.007983	0.0000637
Third order	0.00008132	0.86955	−0.009302	0.00008653

**Table 3:** Analysis table of GC-MS detection results of water samples before oxidation treatment.

Organic Substance	Percentage (%)	Estimated Concentration (mg/L)	Molecular Formula	C	H	O	N	P	Si	Molecular Mass	COD Conversion Factor
2-([1,1′-Biphenyl]-2-aryloxy)ethanol	44.93	19.41	C_14_H_14_O_2_	14	14	2				214	2.46
Dodecamethylcyclohexasiloxane	1.62	0.7	C_12_H_36_O_6_Si_6_	12	36	6			6	445	1.29
Cycloheptasiloxane, tetramethyl	1.53	0.67	C_14_H_42_O_7_Si_7_	14	42	7			7	519	1.29
5-Hydroxy-2,4-di-tert-butylphenyl valerate	2.01	0.8	C_19_H_30_O_3_	19	30	3				306	2.61
o-Hydroxybiphenyl	5.2	2.24	C_12_H_10_O	12	10	1				170	2.63
Hexadecamethylcyclooctasiloxane	1.08	0.47	C_16_H_48_O_8_Si_8_	16	48	8			8	593	1.29
N-Tetrahydro-2-furanylmethyl-1-naphthamide	1.64	0.7	C_19_H_27_NOSi	19	27	1	1		1	255	3.16
1 Acenaphthenol	24.01	10.37	C_12_H_10_O	12	10	1				170	2.63
Oximethoxyphenyl	2.77	1.19	C_8_H_9_NO_2_	8	9	2	1			151	1.96
Triphenylphosphine oxide	0.9	38.8	C_18_H_15_OP	18	15	1		1		278	2.44
Ethyl 2.4.5-trimethoxyphenylpropionate	3.4	1.47	C_14_H_20_O_5_	14	20	5				268	1.97
N-(1-[1,1′-Biphenyl]-2-ethylidenemethyl)	4.71	2.03	C_15_H_15_N	15	15		1			209	2.87
look for a draw (chess)	93.8	43.20									

**Table 4:** Analysis table of GC-MS detection results of water samples after oxidation treatment.

Organic Substance	Percentage (%)	Estimated Concentration (mg/L)	Molecular Formula	C	H	O	N	P	Si	Molecular Mass	COD Conversion Factor
2-([1,1′-Biphenyl]-2-yloxy)ethanol	46.96	5.07	C_14_H_14_O_2_	14	14	2				214	2.46
Oximethoxyphenyl	2.04	0.22	C_8_H_9_NO_2_	8	9	2	1			151	1.96
Dodecamethylcyclohexasiloxane	0.49	0.05	C_12_H_36_O_6_Si_6_	12	36	6			6	445	1.29
Cycloheptasiloxane, tetramethyl	2.52	0.27	C_14_H_42_O_7_Si_7_	14	42	7			7	519	1.29
5-Hydroxy-2,4-di-tert-butylphenyl valerate	0.63	0.07	C_19_H_30_O_3_	19	30	3				306	2.61
o-Hydroxybiphenyl	4.6	0.5	C_12_H_10_O	12	10	1				170	2.63
Hexadecamethylcyclooctasiloxane	1.28	0.14	C_16_H_48_O_8_S_i8_	16	48	8			8	593	1.29
N-Tetrahydro-2-furanylmethyl-1-naphthamide	1.19	0.13	C_19_H_27_NOSi	19	27	1	1		1	255	3.16
1 Acenaphthenol	23.97	2.59	C_12_H_10_O	12	10	1				170	2.63
Triphenylphosphine oxide	0.7	0.08	C_18_H_15_OP	18	15	1		1		278	2.44
Ethyl 2.4.5-trimethoxyphenylpropionate	4.02	0.43	C_14_H_20_O_5_	14	20	5				268	1.97
N-(1-[1,1′-Biphenyl]-2-ethylidenemethyl)	3.3	0.36	C_15_H_15_N	15	15		1			209	2.87
look for a draw (chess)	91.7	10.8									

**Table 5:** Distribution ratio of different ozone catalyst impregnation solutions.

Serial Number	Mn(NO_3_)_2_	Fe(NO_3_)_3_)-9H_2_O	Cu(NO_3_)_2_)-3 H_2_O	Ce(NO_3_)_3_)-6H_2_O
1	4wt%			
2		4 wt%		
3			4 wt%	
4				4 wt%
5	2 wt%	1 wt%		
6	2 wt%	2 wt%		
7	2 wt% 2 wt%		1 wt%	
8	2 wt%		2 wt%	
9	2 wt%			1 wt%
10	2 wt%			2 wt%
11	1 wt%		1 wt%	1 wt%
12	1 wt%		2 wt%	1 wt%
13	1 wt%		1 wt%	2 wt%
14	2 wt%		1 wt%	2 wt%
15	2 wt%		2 wt%	1 wt%
16	2 wt%		2 wt%	2 wt%
17	1 wt%		2 wt%	2 wt%

## Data Availability

The raw data supporting the conclusions of this article will be made available by the authors on request.
